# Central neural circuits and their associated mechanisms of inter-organ crosstalk

**DOI:** 10.3389/fcell.2026.1830768

**Published:** 2026-06-29

**Authors:** Lerui Kang, Jasmine Lu Salas-Hernandez, Ruoshi Xu

**Affiliations:** State Key Laboratory of Oral Diseases & National Center for Stomatology & National Clinical Research Center for Oral Diseases, West China Hospital of Stomatology, Sichuan University, Chengdu, Sichuan, China

**Keywords:** central nervous system, cognitive function, homeostasis, interoception, metabolic diseases, neurological disorders, therapeutic targets

## Abstract

The central nervous system (CNS), which comprises the brain and spinal cord, serves as the core regulatory hub for maintaining homeostasis and coordinating diverse physiological functions. These functions include interoception, cognition, and social behavior. The intricate architecture and extensive connectivity of the CNS enable integration of multi-system responses, thereby ensuring bodily stability and adaptability. The CNS exerts profound regulatory influences on fundamental life-sustaining processes and higher-order cognitive and social functions. This is mediated by distinct neural circuits and molecular mechanisms. CNS dysfunction is strongly associated with the pathogenesis of neurological disorders (Alzheimer’s disease, Parkinson’s disease, stroke and epilepsy), metabolic diseases (obesity and diabetes), and cardiovascular conditions (hypertension and atherosclerosis). Given the pivotal role of the CNS in both physiological regulation and disease progression, a comprehensive understanding of CNS mechanisms is critical for identifying therapeutic targets. This review systematically examines the regulatory functions of the CNS in physiology and disease. Moreover, this review analyzes the underlying molecular and circuit-level mechanisms, and discusses potential therapeutic strategies. By elucidating the systemic interactions of the CNS, this study aims to highlight its potential as a target for innovative interventions in disease prevention, diagnosis, and treatment.

## 1 Introduction

The central nervous system (CNS) is composed of the brain and spinal cord. It is the core regulatory hub for maintaining homeostasis in the internal environment and coordinating complex physiological functions. These functions include interoception (Chen et al., 2017; Mayer, 2011; Levinthal and Strick, 2020), cognition (Song et al., 2024; Shi et al., 2024; Hu et al., 2023; Song and Hu, 2024), and social behavior (Cao et al., 2025; Walsh et al., 2023; Yang et al., 2023). The CNS precisely controls life support processes through multi-level neural circuits and molecular mechanisms (Hosang et al., 2024; Veerakumar et al., 2022) and plays an irreplaceable role in integrating peripheral signals and driving adaptive behavioral output (Deisseroth, 2014; Wang and Chang, 2024). CNS dysfunction is closely related to the pathogenesis of neurodegenerative diseases (ND) (Zhang et al., 2023; Doifode et al., 2021; Martin, 1999), metabolic diseases (Abel et al., 2024; Sewaybricker et al., 2023), cardiovascular diseases (Veerakumar et al., 2022; Li G. et al., 2025), and other major diseases. Therefore, understanding the regulatory principles of the CNS is of great basic and clinical importance.

Many studies have focused on the brain regions (Levinthal and Strick, 2020; Phillips et al., 2019), molecules (Froemke and Young, 2021; Crane et al., 2020; Casado-Sainz et al., 2022; Chen et al., 2024; Dölen et al., 2013; Okaty et al., 2019) and cell types (Dienel, 2019; Xu et al., 2022) involved in specific functions. However, the mechanism of how the CNS uses a relatively limited variety of molecules and cells to achieve infinite functional states remains unclear. Most studies focus on single targets or isolated pathways. Hence, the dynamic coordination at the molecular, cellular, and systemic levels in CNS regulation remains unclear. Furthermore, an integrated theoretical framework explaining the mechanism of local regulatory network failures gradually evolving into systemic failures in diseased states is lacking.

In response to the above issues, this review focuses on the three core levels of CNS regulation. At the molecular level, the signal function is encoded by spatial localization (Macgillavry et al., 2013; Ferreira et al., 2020), temporal dynamics (Booker et al., 2017; Ren et al., 2022; Gonzalez-Hernandez et al., 2024), and molecular combinations (Gamo et al., 2015; Arnsten, 2011), which is a process called molecular cross-coding. This determines the direction and strength of synaptic plasticity. At the cellular level, glial cells constitute a complementary information processing system closely related to neurons. It is written directly into circuit parameters through activity-dependent phagocytosis (Paolicelli et al., 2011; Pereira-Iglesias et al., 2025) and myelin plasticity (Taylor and Monje, 2023). At the system level, the cross-organ network integrates peripheral and central information through a three-layer architecture of molecular messenger (Janeiro et al., 2023; Hsiao et al., 2013), cell carrier (Kaelberer et al., 2018) and neural pathway (Xie et al., 2022). These mechanisms suggest that the functional realization of the CNS depends on multiple coding rules from the molecular level to the systemic level. Dissecting this set of rules in depth is key to understanding how it works. Disease may correspond to the local disruption of these coding rules with regulatory dimensions. Accordingly, this review proposes a theoretical model of multi-level and cross-scale combinatorial intervention. The core idea is that treatment should shift from targeting a single pathological marker to repairing a multi-dimensional microenvironment. This will enable a paradigm transition from symptom control to disease modification through space-time specific strategies. Overall, this strategy provides a new theoretical reference for the mechanism analysis and precision treatment of the nervous system and related diseases.

## 2 Regulation of physiological functions by the CNS

### 2.1 Interoception

Interoception is the core process by which the CNS perceives, integrates, and interprets internal state signals of the body. This process constitutes the physiological basis for the body to maintain homeostasis and form self-awareness (Chen et al., 2021). As a superlative integrator of interoceptive information, the CNS translates dispersed physiological signals into unified somatic perception, and coordinates systemic adaptive responses accordingly.

**FIGURE 1:**
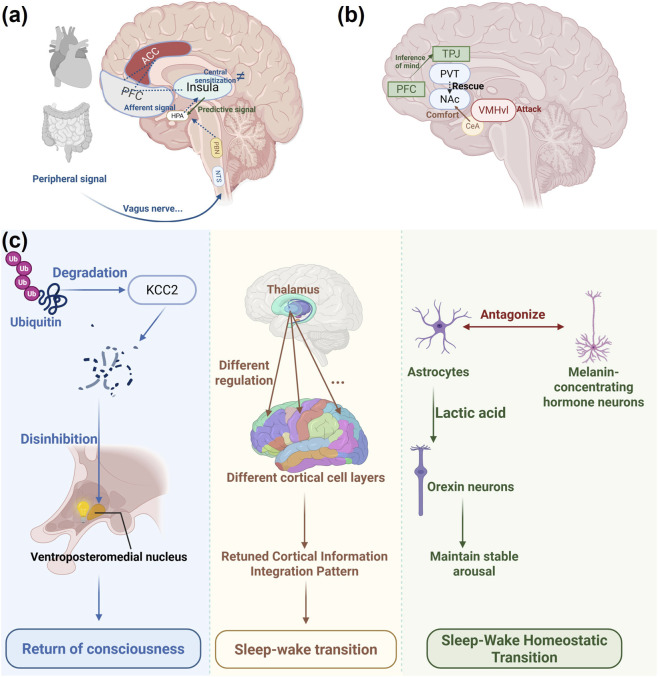
The integrated regulation of physiological functions by the CNS. **(a)** Interoception: peripheral signals relay through the brainstem and thalamus and then converge on higher cortex such as the insula. The insula and prefrontal cortex generate predictive signals and the afferent signals are compared in real time, and the mismatch results in central sensitization. **(b)** Social behavior: specialized neural circuits provide behavioral templates. **(c)** Cognition and consciousness: ubiquitin-mediated degradation of KCC2 in the VPM drives consciousness recovery. Hierarchical regulation of the cortex by medullary nuclei enables arousal transition. Hypothalamic orexin antagonizes MCH to maintain homeostasis.

The transmission of interoceptive signals follows a clear hierarchical neural pathway (Levinthal and Strick, 2020; Phillips et al., 2019) (Figure 1a). First, peripheral signals are received in the primary afferent layers within the spinal dorsal horn and nucleus tractus solitarii. Subsequently, initial processing and filtering occur in the relay integration layer within the parabrachial nucleus and thalamus. Information finally converges at the higher-order integration and consciousness layers. Among them, the insular cortex, as the core hub, cooperates with the prefrontal cortex, anterior cingulate cortex, amygdala, and other brain areas to produce conscious body sensations, emotional experiences, and cognitive assessments.

The CNS does not passively regulate interoception but rather conforms to a theoretical model called predictive coding (Friston and Kiebel, 2009). Higher-order brain regions continuously generate internal predictive signals regarding body states based on previous experiences. These predicted signals are compared with and calibrated against actual incoming signals from the relay layer (Nara et al., 2025). A long-term mismatch between prediction and input may lead to dysregulation of interoception, which mistakes normal physiological signals for threats. This is known as central sensitization, in which the CNS overreacts to normal stimuli (Velasco et al., 2024). Therefore, the essence of precise interoception regulation by the CNS is to continuously optimize and revise this internal prediction model.

Dysregulation of these regulatory mechanisms may constitute a common pathological basis for various diseases. This renders interventions targeting interoceptive pathways or predictive models potential emerging therapeutic strategies.

### 2.2 Cognition and awareness

Cognition and consciousness are high-level outputs of the CNS that are achieved by dynamically coordinating peripheral physiological signals with intrinsic neural homeostasis (Redinbaugh et al., 2020). Traditional studies have focused on locating specific brain regions (Chen et al., 2018; Cohen et al., 2005; Baker et al., 2016). However, cognitive clarity and consciousness level are closely related to whether the CNS can accurately interpret the whole body state and effectively maintain the balance of its internal network (Berntson and Khalsa, 2021; Klein et al., 2021; Damasio and Damasio, 2024) (Figure 1c).

The CNS continuously integrates signals from the periphery to transform dispersed physiological information into a unified cognitive state. In particular, cardiovascular, respiratory, and metabolic signals, which are processed at all levels of the CNS, converge in the thalamus, subcortical nuclei, and higher-order cortex. Individual neurons in the thalamus and subthalamic nucleus encode heart rate and respiratory rhythms, thereby suggesting an integrated role for these nuclei in body-brain communication (De Falco et al., 2024). The states of systems, such as skeletal (Han et al., 2018) and immune (Piehl et al., 2022; Gate et al., 2020) systems, also affect cognitive function. The brain may integrate these peripheral states as reference parameters for cognitive models, thereby forming a coordinated cognitive experience.

Maintenance and recovery of consciousness depend on a series of active regulatory processes within the CNS. Ubiquitin-mediated degradation of K^+^-Cl^-^ cotransporters, such as potassium-chloride cotransporter 2, in the ventral posteromedial nucleus of the thalamus (VPM) relieves local neural depression and initiates functional reconstruction of the thalamocortical circuit to drive recovery of consciousness (Hu et al., 2023). Multiple subcortical thalamic nuclei, including the nucleus medullosus, exert differential regulation in different cortical cell layers, thereby regulating the cortical information integration mode and realizing a dynamic transition from sleep to wake (Müller et al., 2023). In addition, orexin neurons in the lateral hypothalamus stabilize arousal through lactate metabolic support provided by astrocytes. These neurons also act in dynamic antagonism with pro-melanin-concentrating hormone neurons to synergistically regulate sleep–wake homeostasis transitions and provide an optimal timing window for cognitive activity (Li P. et al., 2025; Braga et al., 2024). The clarity of cognitive function and awakening of consciousness depend on a complex regulatory network involving multiple brain regions and molecular pathways.

### 2.3 Social behaviors

Social behavior is the output of real-time calculations and the integration of multi-dimensional social information by the CNS. This process relies on the dynamic context-specific regulation of hierarchical neural circuits by evolutionarily conserved molecular systems.

Social behaviors involve highly specialized neural circuits as the anatomical basis for their execution (Figure 1b). The pathway from glutamatergic neurons in the paraventricular nucleus of the thalamus to the nucleus accumbens shell drives rescue behavior (Cao et al., 2025), and the central amygdala → nucleus accumbens pathway is involved in emotional comfort (Ferretti et al., 2019; Rogers-Carter et al., 2019; Wu and Hong, 2022). Moreover, the medial prefrontal cortex → temporoparietal junction pathway supports psychological inference (Yang et al., 2022). These pathways collectively constitute a parallel computing network for processing different social dimensions. Notably, mirror neurons in the ventromedial hypothalamic nucleus (VMHvl) are activated both when an animal initiates aggressive behavior on its own and when observing a similar aggressive behavior (Yang et al., 2023; Yang et al., 2017). From an evolutionary perspective, this suggests that the brain may utilize the same concise code to process the execution and recognition of social behavior. This mechanism enables individuals to realize rapid recognition and adaptive responses (Yang et al., 2023).

However, fixed circuits cannot account for behavioral flexibility in comparison with context dependence, which requires the dynamic regulation of molecular systems. The core function of neurotransmitters, such as oxytocin (Froemke and Young, 2021; Crane et al., 2020), dopamine (Casado-Sainz et al., 2022; Chen et al., 2024), serotonin (Dölen et al., 2013; Okaty et al., 2019) is to precisely modulate specific neural circuit properties. These properties include synaptic efficacy, neuronal excitability, and network oscillations, which are the rhythmic synchronous activity of neuronal ensembles. These modulations occur in accordance with an individual’s internal state, such as stress and hormone levels, and external social contexts. For example, dopamine enhances the motivational value of prosocial behavior in reward-related circuits (Kopec et al., 2019). Serotonin may modulate thresholds for aggressive responses in amygdala circuits (Okaty et al., 2019; Audero et al., 2013). The same molecule has radically different effects on different brain regions or in different behavioral contexts, thereby conferring rich adaptive outputs to fixed circuits.

Future research should quantitatively analyze the dynamic clustering of molecular signals and neural cluster activity in natural social interactions (Walsh et al., 2023) and the resulting adaptive social decisions using real-time synchronous recording technology of behavior and neural activity.

## 3 The role of the CNS in the occurrence and development of diseases

The normal operation of the aforementioned physiological functions depends on the precise coding rules of the CNS at the molecular, cellular, and network levels. When these rules are disrupted in specific dimensions, physiological regulation emerges as a pathological process. In the following section, neurodegenerative, metabolic, and cardiovascular diseases are used as examples to demonstrate the effects of different levels of coding disruptions on disease occurrence and progression.

### 3.1 Mechanisms of CNS regulation of neurodegenerative diseases

**FIGURE 2:**
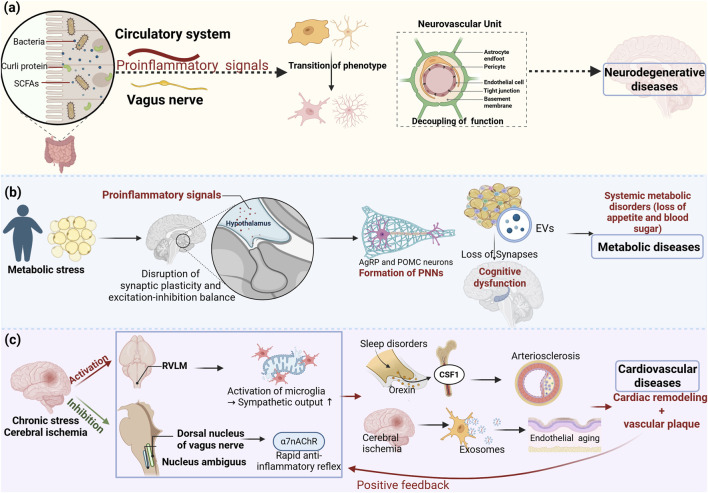
Schematic representation of the core mechanisms of CNS regulation of diseases of the three major systems. **(a)** Abnormal peripheral signals are transmitted to the CNS via the vagus nerve and circulation, driving glial cells from a protective to a pathologic phenotype and leading to the uncoupling of the neurovascular unit (NVU), ultimately triggering the collapse of neural networks. **(b)** Metabolic stress induces neuroinflammation in the hypothalamus. Abnormal remodeling of the perineuronal network (PNN) around neurons solidifies reversible functional abnormalities into structural lesions. This solidification establishes a positive feedback loop with peripheral signals such as adipose tissue exosomes, driving disease progression. **(c)** Chronic stress or cerebral ischemia redirects central regulation from the rapid anti-inflammatory reflex of the brainstem to neuroinflammation and sympathetic hyperactivity in the rostral ventrolateral medulla (RVLM), thereby driving cardiovascular remodeling and atherosclerosis through positive feedback along the brain-vascular axis.

The CNS actively participates in, and profoundly affects, the progression of NDs by integrating peripheral signals, coordinating internal glial cell responses, and maintaining neurovascular homeostasis (Figure 2a). Disease progression is a stepwise breakdown of a complex regulatory network.

Considering that the CNS serves as an integration hub of systemic risk signals, CNS dysfunction is an important starting point for disease. Gut–brain axis studies indicate that the dysbiosis of the intestinal microbiota (Gha et al., 2016) and its metabolic products (short-chain fatty acids (Cryan et al., 2020)) and the presence of abnormal proteins (Curli protein (Martin, 1999)) continuously transmit pro-inflammatory and pro-aggregation signals to the CNS through the circulatory system and the vagus nerve. This shapes a central microenvironment unfavorable for neuronal survival in diseases such as Alzheimer’s disease (AD) (Zhang et al., 2023; Doifode et al., 2021) and Parkinson’s disease (PD) (Martin, 1999) prior to substantial neuronal loss (Singh et al., 2021). This suggests that persistent abnormal signals from peripheral organs are specifically sensed and integrated by the CNS to drive or exacerbate the central pathology.

Glial cells within the CNS play an important role in the maintenance of homeostasis. Dysregulation of their response is considered an important amplifier of disease progression. The functions of microglia and astrocytes in NDs exhibit phasic heterogeneity (phenotypes vary with disease progression) (Dienel, 2019; Xu et al., 2022; Block and Hong, 2005; Mahad and Ransohoff, 2003) and regional heterogeneity (glial responses vary in different brain regions) (Serrano-Pozo et al., 2024; Verkhratsky and Nedergaard, 2018). In early stages, glial cells may attempt to protect themselves by engulfing pathological proteins (Block and Hong, 2005; Mahad and Ransohoff, 2003). However, as the disease progresses, persistent abnormal signaling, such as α-synuclein (Williams et al., 2021), drives glial cells into a dysfunctional disease-related state. This state is characterized by synaptic overpruning, ongoing neuroinflammation (Zhao et al., 2024), and reduced nutritional support, such as impaired lactate shuttling by astrocytes (Dienel, 2019; Xu et al., 2022). This shift from protective homeostasis to destructive responses might create a self-reinforcing cycle that accelerates neuronal network dysfunction.

The neurovascular unit (NVU) is a highly integrated multicellular complex composed of neurons, astrocytes, microglia, brain microvascular endothelial cells, pericytes, and the extracellular matrix (ECM). The NVU forms the structural and functional basis of the blood-brain barrier (BBB) (Wang et al., 2026; Kim and Filosa, 2012). Functional decoupling of the NVU is considered a key link in the loss of systemic regulation ability of the CNS and irreversible decline (Kempuraj et al., 2024). In NDs, neuroinflammation and pathological proteins selectively disrupt different functional modules of the NVU (Par et al., 2017). In early disease stages, the NVU may impair the efficiency of amyloid beta clearance by the glymphatic system (Huang et al., 2024; Harrison et al., 2020) or affect local neuromodulator synthesis (Zhu et al., 2022). In late disease stages, this may result in pronounced blood-flow disturbances (Sweeney et al., 2019; Sandsmark et al., 2019). This uncoupling between different functional modules gradually reduces the ability of the brain to maintain homeostasis, remove toxic substances, and resist secondary damage. This eventually collapses large-scale neural network functions.

Future intervention strategies should surpass single neuronal protection or protein clearance and shift from targeting pathology to reestablishing central homeostasis.

### 3.2 Neural circuit mechanisms in metabolic diseases

An important neural mechanism of metabolic diseases is pathological solidification of key neural circuits responsible for energy and glucose homeostasis (Czech, 2017; Mirzadeh et al., 2022) (Figure 2b).

The initiation of pathological solidification may be related to neuroinflammation-induced impairment of circuit plasticity. Under metabolic stress, such as obesity (Klein et al., 2022), neuroinflammation dominates key brain regions such as the hypothalamus (Abel et al., 2024; Emont et al., 2022). Pro-inflammatory signals destroy the molecular environment that maintains neuronal synaptic plasticity and normal excitation–inhibition balance. This results in decreased sensitivity of neural circuits to key metabolic hormones (Lyu et al., 2020).

Persistent neuroinflammation drives abnormal remodeling of ECM components, such as the perineuronal network, including changes in the sulfation pattern and abnormal increases in density (Beddows et al., 2024). This structurally stabilizes the disordered neuronal connectivity patterns and electrical activity properties and may transform reversible functional abnormalities into permanent structural abnormalities. This is thought to be the material basis of pathologic solidification.

A self-reinforcing feedback loop of abnormality may form between the solidified central circuit and the disturbed peripheral organs. In the context of rigid circuit function, exosomes from abnormal adipose tissues carry specific microRNAs and other signals that continuously transmit erroneous instructions to the CNS, thereby reinforcing insulin resistance and energy storage (Wang et al., 2022). This creates a positive feedback loop (Lewis, 2024; Yoon and Diano, 2021) whereby the disease is progressively aggravated.

Future Frontier therapies could focus on eliminating neuroinflammation, targeting the abnormal ECM, and providing correct reprogramming signals to repair metabolic regulatory functions at the systemic level.

### 3.3 Mechanisms of CNS regulation of cardiovascular disease

The CNS maintains cardiovascular homeostasis through a dynamically integrated network with a hierarchical structure and situational dependence (Figure 2c).

In response to well-defined and immediate signals, the CNS invokes dedicated microloops at the brainstem level to perform efficient and specific reflexes. For example, the direct cholinergic pathway from the dorsal nucleus of the vagus nerve to the heart achieves rapid local anti-inflammation by activating alpha7 nicotinic acetylcholine receptors (α7nAChR) on myocardial macrophages (Li G. et al., 2025). Neuronal clusters coordinating heart rate and bronchoconstriction in the nucleus may ensure the precise execution of cooperative defense behaviors, such as the diving reflex (Veerakumar et al., 2022). These circuits are relatively independent in function and contribute to precise and rapid regulation, which is important for maintaining basal homeostasis and executing innate defenses.

During persistent or complex challenges, structures such as the hypothalamus and limbic system are extensively recruited, and the regulatory core shifts to long-term multi-system functional readjustment. Under chronic stress, neural–immune–metabolic interactions occur in the rostral ventrolateral medulla. For example, microglial activation damages mitophagy, thereby aggravating neuronal excitability and sympathetic output (Zhang et al., 2020). This is considered a form of dysregulated adaptive program. Furthermore, the CNS coordinates the adaptive structural remodeling of the heart to the pressure load by remotely regulating the function of splenic immune cells (Perrotta et al., 2025). This suggests that during the chronic phase, CNS regulatory outputs are diverse, cross-organ and deeply embedded in the immune-metabolic dialogue.

Changes in the physiological and pathological states of the brain actively alter the peripheral cardiovascular environment through neurohumoral pathways. For example, sleep disorders promote monocyte generation and accelerate atherosclerosis through the hypothalamic orexin–bone marrow colony stimulating factor 1 axis (Mcalpine et al., 2019). After cerebral ischemia, microglia-derived exosomes deliver neurogenic locus notch homolog protein 1 ligands to peripheral blood vessels through the circulatory system, thus continuously driving endothelial inflammation and senescence (Liu et al., 2024). These findings suggest the bidirectional nature of the brain–vascular axis.

Future interventions for cardiovascular diseases should be updated based on this dynamic hierarchical model. Specifically, interventions should shift from the management of downstream symptoms to fundamental treatment of upstream systemic regulation.

## 4 Recent progress in the mechanisms of CNS regulation

The aforementioned disease mechanisms are mostly described at the systemic or organ level. However, understanding why these pathological processes are pervasive and intractable requires knowledge of basic molecular, cellular, and network-coding principles.

### 4.1 Molecular crosstalk in the CNS regulation

**FIGURE 3:**
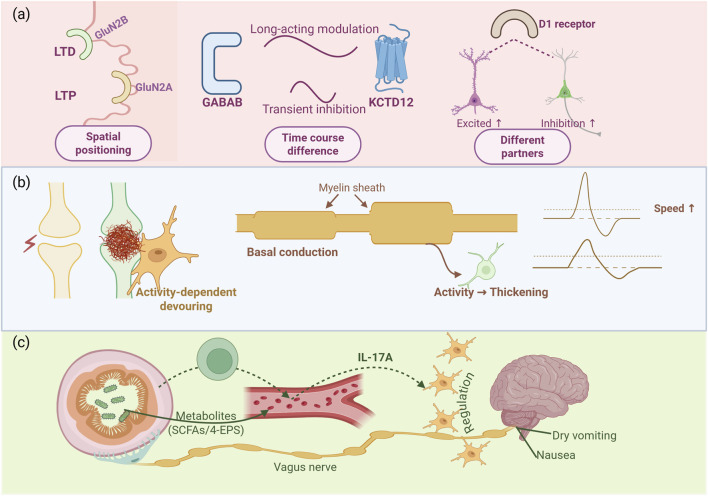
Multi-level mechanisms of CNS regulation. **(a)** At the molecular level: signal functions are encoded by spatial localization, time-course differences, and molecular combinations, which determine the direction and strength of synaptic plasticity. **(b)** At the cellular level: microglia regulate synaptic weight through activity-dependent phagocytosis, and oligodendrocytes regulate conduction velocity through myelin plasticity, and these processes together constitute a second information processing system complementary to neurons. **(c)** Network level: the gut-brain axis integrates peripheral and central information through three parallel pathways, namely, metabolites, immune cells and the vagus nerve.

The types of molecules available in the CNS are relatively limited. However, these molecules must support an infinite number of functional states that range from synaptic plasticity to complex behavioral outputs. This contradiction may partly be resolved by the multi-dimensional interaction between existing molecular signals to achieve the spatiotemporal-specific output of a function in a specific neural circuit context. Simply put, this refers to the ultimate production of a specific physiological effect at a specific synaptic location and within a precise time window. This is summarized into three interaction dimensions: spatial encoding, temporal coding, and combinatorial coding (Figure 3a).

Spatial encoding is based on differences in the nanoscale distribution of receptor molecules of the same class on the surface of neurons. These differences greatly affect the direction of their signal output (Macgillavry et al., 2013; Ferreira et al., 2020). For example, the distribution of the N-methyl-D-aspartate receptors GluN2A and GluN2B in the postsynaptic dense region determines the local spatial characteristics of calcium signals entering the cytoplasm and specifically directs long-term potentiation or inhibition (Iacobucci and Popescu, 2019; Dupuis et al., 2023).

In temporal coding, the temporal dimension is considered to be a key transition axis from quantitative to qualitative changes in molecular signals. The same pathway generates completely different physiological instructions owing to differences in the time course. The kinetics of the gamma-aminobutyric acid type B receptor binding to its co-protein (potassium channel tetramerization domain containing 12) is a key factor affecting the time course of G-protein signaling. Rapid dissociation results in transient inhibition, whereas sustained binding mediates long-lasting modulation (Booker et al., 2017; Ren et al., 2022; Gonzalez-Hernandez et al., 2024).

In combinatorial coding, individual receptor proteins acquire functional diversity by coupling with effector proteins in different cell types and membrane microdomains. Dopamine D1 receptors are coupled to different downstream signaling pathways in cortical pyramidal neurons and interneurons and exert opposite regulatory effects on synaptic plasticity (Gamo et al., 2015; Arnsten, 2011).

The molecular pathology of neurological diseases is the local disruption of coding rules. Future therapeutic strategies may shift from targeting molecular entities to regulating the interactions between molecules.

### 4.2 Cellular mechanisms in the CNS regulation

The microglial and oligodendrocyte lineages are complementary information-processing systems that are closely related to neurons (Figure 2b). Neurons encode information via action potentials and synaptic transmission. In contrast, glial cells regulate the connection strength of neural circuits and the temporal characteristics of signal transmission by selectively executing phagocytic events (Paolicelli et al., 2011; Pereira-Iglesias et al., 2025) and activity-dependent adjustments of myelin structure (Taylor and Monje, 2023).

Complement-dependent phagocytosis of synapses by microglia is not passive cleaning behavior (Eyo and Molofsky, 2023). Microglia selectively engulf synapses with low-frequency activity or weak electrical input, while avoiding synapses with strong input (Devries et al., 2024; Faust et al., 2021). This suggests that phagocytic events are computational processes that downregulate the strength of synaptic connections based on local synaptic activity history. This process exhibits high input specificity and spatial and temporal precision, and its regulatory mechanism may not be entirely determined by neuronal electrical activity patterns.

Neuronal activity regulates both oligodendrocyte precursors and mature oligodendrocytes. The latter dynamically adjusts myelin sheath thickness and internodal length, thereby altering the action potential conduction velocity (Steadman et al., 2020). This suggests that the time delay in neural circuits may be plastic, and myelin may be an empirically modified regulator of conduction delay, thereby affecting the time taken for an action potential to propagate along axon (Noori et al., 2020). The temporal coding accuracy of neural circuits is partly determined by the structural plasticity of glial cells.

Hence, the cytopathology of neuropsychiatric disorders can be understood from this perspective. Excessive synapse clearance by microglia in AD is not a simple reactive process. Instead, the synaptic pruning function of microglia is abnormal owing to the disorder of microenvironmental signals (Ding et al., 2021). Abnormalities in myelin architecture in white matter lesions (Nunez and Srinivasan, 2014) reflect oligodendrocyte damage and may also represent a systemic deviation in the regulation of conduction delays. Future therapeutic strategies may shift from nonspecific inhibition of glial cell activity to the modification of decision-making mechanisms and thresholds to trigger structural changes in response to microenvironmental signals.

### 4.3 Network mechanisms in the CNS regulation

The CNS achieves dynamic perception, signal integration, and feedback regulation of physiological states through a multi-level network in various organs throughout the body. An increasing number of studies have been conducted on the gut–brain axis, brain–heart–spleen axis, and liver–brain–pancreas axes. This suggests that CNS regulatory networks may have some universal characteristics.

The brain–gut axis is a dynamic network consisting of three factors: molecular messengers, cellular carriers (signal-carrying immune cells), and neural pathways (Figure 3c). These factors ultimately realize bidirectional information integration. In the molecular layer, gut microbial metabolites enter circulation as first messengers. Short-chain fatty acids regulate the development and maturation of microglia and immune functions through G protein-coupled receptors and histone deacetylase inhibition mechanisms (Kim, 2021; Yu et al., 2014; Park and Kim, 2021). Indole derivatives produced during tryptophan metabolism act as endogenous ligands for aryl hydrocarbon receptors to regulate the inflammatory response thresholds of astrocytes and microglia (Rothhammer et al., 2019; Rothhammer et al., 2016). Some pathogenic metabolites, such as trimethylamine N-oxide (Janeiro et al., 2023) and 4-ethylphenyl sulfate (Hsiao et al., 2013; Wang et al., 2023), may disrupt microglial homeostasis and aggravate neuroinflammation and myelin sheath injury. In the cellular layer, enteroendocrine cells in the intestinal epithelium release hormones, such as cholecystokinin and peptide YY, through endocrine pathways and form direct synaptic connections with vagal nerve terminals by using glutamate as a transmitter to achieve millisecond signal transmission (Kaelberer et al., 2018). Gut-derived immune cells also constitute a cellular channel type. After abnormal activation in the intestinal inflammatory microenvironment, gut microbiota-specific cluster of differentiation 4 T cells migrate to the CNS and are reactivated by host protein antigens through a molecular mimicry mechanism. This produces granulocyte-macrophage colony-stimulating factor, interferon-γ, and interleukin-17A, thereby activating microglia and causing nerve damage (White et al., 2025). In the nerve layer, the vagus nerve is the most direct high-speed pathway, and its afferent fibers transmit local events from the gut to the nucleus tractus solitarius and area postrema of the medulla oblongata within milliseconds. For example, after sensing enterotoxins or chemotherapeutic drugs, enterochromaffin cells release 5-hydroxytryptamine (5-HT), which activates vagal afferent fibers that express 5-HT receptors. This subsequently activates tachykinin-expressing neurons in the dorsal vagal complex of the medulla oblongata, which regulates nausea and retching via two parallel projections (Xie et al., 2022).

Additional innovative networks are constantly being revealed. In the brain–heart–spleen axis, the CNS supports cardiac structural remodeling under cardiac stress and overload by remotely regulating the function of immune cells in the spleen (Perrotta et al., 2025). In the liver–brain–pancreas axis, a neural circuit promotes the compensatory proliferation of pancreatic β cells in obesity (Yamamoto et al., 2017). In the bone–brain axis, osteocalcin secreted by bone cells crosses the BBB and activates G protein-coupled receptor 158 in the hippocampus, thereby regulating learning and memory (Ha et al., 2025).

Based on the above findings, the network regulation of the CNS presents several common characteristics, namely, multi-level collaborative coding and cross-organ joint scheduling. Future treatment strategies should shift from single-target intervention to targeting the key hub in the regulatory network. This will enable reconstruction of information flow by restoring the signal transmission timing and strength between different levels. Moreover, future treatment should focus on restoring dynamic cooperation between the molecular, cellular, and neural layers, thereby inducing homeostatic repair at the system level.

## 5 The support of existing therapeutic interventions for the combination treatment model

Coding rules at the molecular, cellular, and network levels constitute the fundamental principles of CNS regulation. Therefore, an ideal therapeutic strategy should go beyond targeting individual molecules or cells and instead restore the coding environment at multiple levels. This section demonstrates how existing interventions support this multi-level combination model to varying degrees, thereby deriving the conceptual framework of sequential combination intervention.

### 5.1 Gut-brain axis intervention

The gut–brain axis intervention provides initial support for the proposed multi-level combinatorial conceptual framework. The interventions of butyrate (gut microbiota metabolite) and strain supplementation show promising combinatorial potential owing to their different sites of action.

Butyrate is one of the main short-chain fatty acids produced by the gut microbiota from dietary fiber. It shows vital multi-level characteristics in CNS regulation. In the molecular layer, butyrate acts as a histone deacetylase inhibitor, thereby producing anti-inflammatory effects (Wu et al., 2008; Rahmani et al., 2025). In the cellular layer, it inhibits microglial activation and pro-inflammatory factor production through the protein kinase B/nuclear factor-kappa B pathway, thus improving pathological damage (Lv et al., 2024). At the network level, synergistic effects at the molecular and cellular levels are ultimately reflected in behavioral improvement and neurological protection.

The abundance of *Akkermansia muciniphila* is markedly reduced in the intestines of PD mice (Qiao et al., 2024). Exogenous supplementation with this strain inhibits microglia-mediated neuroinflammation, protects dopaminergic neurons, and improves motor function by increasing the butyrate content in the intestine and brain (Xu et al., 2025). In addition, *A. muciniphila* reduces astrocyte activation, alleviates inflammation, and regulates gut microbiota composition (Xi et al., 2025).

A complementary relationship may exist between the anti-inflammatory effects of butyrate and the optimization of the intestinal barrier by *A. muciniphila*. Specifically, *A. muciniphila* creates a favorable microenvironment for the colonization and metabolism of butyrate-producing bacteria by strengthening the intestinal mucus layer (Shuoker et al., 2023). Consequently, butyrate-mediated histone deacetylase 1 inhibition alters host epigenetic status and provides a molecular basis for gut–brain axis network remodeling after *A. muciniphila* colonization (Mabry et al., 2025). This suggests a potential combination strategy based on collaborative patterns and sequential adaptation. At different disease stages, butyrate is first used to rapidly control CNS inflammation, followed by *A. muciniphila* to reinforce and maintain a healthy circuit state within the microbiota network over the long term. This type of targeted, hierarchical, multi-target intervention is an important attempt to shift towards the multi-level and cross-scale combinatorial conceptual model that is the focus of this review.

### 5.2 Metabolic disease interventions

Glucagon-like peptide-1 (GLP1) receptor (GLP1R) agonists (GLP1RA) are first-line drugs for type 2 diabetes and obesity and rapidly regulate neural circuit functions. In the molecular layer, GLP1RAs regulate feeding through protein kinase A-mediated phosphorylation of key mechanistic target of rapamycin complex 1 proteins (Le et al., 2023). In the cellular layer, tirzepatide, a dual-receptor agonist of GLP1/glucose-dependent insulinotropic polypeptide (GIP), markedly inhibits hypothalamic microglial activation and pro-inflammatory gene expression. This effect is independent of weight loss (Marinho et al., 2026). GLP1RAs regulate feeding behavior by activating GLP1R neurons in multiple brain regions, such as the dorsomedial hypothalamic nucleus and nucleus tractus solitarii, thereby enhancing synergistic activity between these brain regions (Cao and Tong, 2024; Kim et al., 2024). These effects have a rapid onset, are dependent on continuous dosing, and are prone to rebound after discontinuation.

Fibroblast growth factor (FGF) 1 analogs may exert long-lasting effects by inducing ECM remodeling. In a rat model of type 2 diabetes mellitus, a single intraventricular injection of FGF1 induced durable glycemic remission. This effect stems from FGF1 promoting the assembly and remodeling of perineuronal network (PNN), which are reticular ECM structures surrounding specific neuronal cell bodies, in the arcuate nucleus. This physically stabilizes the healthy state of circuit connections (Alonge et al., 2020). Moreover, FGF1 induced the transformation of a subset of Agouti-related peptide neurons in the arcuate nucleus from a pathologically hyperactive state to a hypoactive wild-type state. This was accompanied by the upregulation of PNN components and cooperation of glial cells (Aalling et al., 2026). This enables multi-dimensional durable repair encompassing molecular signaling, cellular state, and ECM structure.

GLP1RAs provide rapid and reversible circuit reset, whereas FGF1 provides slow and durable structural solidification. Hence, these molecules complement each other in terms of onset speed and durability of action. GLP-1 and FGF21 were fused into a bifunctional molecule to achieve synergistic effects with a single administration. This supports the feasibility of this combination concept. Therefore, future studies should aim to rapidly reset the loop function with GLP1RAs and solidify the PNN structure with FGF1/FGF21 analogs. This will advance treatment from symptom control to long-lasting disease modification.

### 5.3 Cardiovascular disease interventions

Vagus nerve stimulation (VNS) selectively activates choline acetyltransferase-positive neurons in the dorsal motor nucleus of the vagus nerve. Acetylcholine is released from the vagus nerve and acts on α7nAChRs on cardiac C-C chemokine receptor-like 2 positive (CCRL2^+^) macrophages. This upregulates nuclear factor erythroid 2-related factor 2 (transcription factor), suppresses the tumor necrosis factor-alpha response, and blocks the progression of pressure overload-induced heart failure (Li G. et al., 2025; Hoover, 2017). The VNS intervention covers multiple levels: brainstem nerve discharge (network layer), acetylcholine release (molecular layer), macrophage phenotype switching (cell layer), and cardiac function improvement.

The α7nAChR agonist EVP 6124 effectively inhibits the inflammatory response of CCRL2^+^ macrophages and improves heart failure (Li G. et al., 2025). Moreover, GTS-21 (selective partial α7nAChR agonist) supports the cardioprotective effect of the α7nAChR pathway in a rat model of cardiopulmonary resuscitation (Kong et al., 2018). Furthermore, α7nAChR agonists accurately target key molecular nodes but cannot reproduce the broad VNS-induced regulation of the sympathetic and parasympathetic nervous systems.

The advantage of VNS lies in the simultaneous activation of the three-layer signal transduction chain from network to molecule to cell, while α7nAChR agonists precisely anchor the core nodes of the chain. For heart failure subtypes, such as heart failure with preserved ejection fraction, transcutaneous auricular vagus nerve stimulation (taVNS) can be used to rapidly activate parasympathetic tone. This initiates a three-tiered defense mechanism comprising molecular anti-inflammatory actions, cellular phenotypic switching, and network regulation. Subsequently, an α7nAChR agonist is introduced to maintain and protect the sustained activation of the key α7nAChR pathway (Huo et al., 2026). This combination leverages the advantages of VNS integration and subsequent maintenance of targeted agonists to translate short-term nerve stimulation effects into long-lasting treatment regimens. This provides a promising translational path for multi-level and cross-scale combinatorial intervention in cardiovascular diseases.

### 5.4 Integration and innovation: Sequential combination intervention

These intervention examples across various functional axes suggest that a single intervention focuses on one domain, even when it has multi-target effects. Therefore, future strategies should emphasize integrating interventions with different mechanisms into sequential combination interventions. Specifically, different interventions should be used in a specific sequence to achieve the best synergistic effect.

This strategy is gaining momentum and is supported by research advances. In the metabolic field, the GLP1/FGF21 dual-target fusion protein (HEC88473) completed its first-in-human clinical study and advanced to Phase II, thereby demonstrating the functional combination of single-molecule mechanisms (Zhang et al., 2025). The GLP1/GIP/FGF21 triple agonist MWN105 has also been clinically developed (Song, 2025). In ND research, cell type-targeted multi-target drug discovery strategies have been applied to AD, and combination therapies targeting both neurons and glial cells have markedly improved cognitive deficits, with superior performance over any single-target agent (Li Y. et al., 2025).

The sequential combination intervention suggests a possible paradigm shift in clinical trial design. In this design, treatment endpoints are not limited to changes in symptom scores or single biomarkers. Instead, the degree and sustainability of the repair of multiple levels of regulatory mechanisms are assessed.

## 6 Conclusions and prospects

This study systematically describes a multi-level, cross-scale, and combined intervention model for CNS regulation. The core idea is that disease treatment should shift from targeting single pathological markers to repairing multi-dimensional microenvironments and circuit dialogues. The essential difference between this conceptual model and traditional strategies is that the spatiotemporal specificity and stage integration of the interventions are emphasized. Effective treatment should be based on the pathological characteristics of the different disease stages, and the timing of coordinated intervention combinations should be optimized.

The systemic repair of the molecular–cell–circuit–system continuum has been emphasized. The normal function of the CNS depends on the coordinated operation of coding rules at multiple levels, from the molecular level to the systemic level. Therefore, effective interventions must cover this continuum rather than target a single node in isolation.

This review proposes a new therapeutic paradigm for repairing coding rules rather than targeting molecular entities. Based on the review findings, the pathological nature of neurological diseases is local disruption of specific coding dimensions. Accordingly, future therapeutic strategies should focus on restoring the correct subcellular localization of receptors, correcting abnormal signal dynamics, reprogramming the functional state of glial cells, and reconstructing multi-level cooperation across organ networks.

This framework has direct implications in the fields of oral and maxillofacial science. In particular, sensory integration, motor control, and pain perception in the oral and maxillofacial regions rely on specific neural circuits between the brainstem and cortex. Furthermore, central sensitization in common diseases may be associated with abnormalities in interoceptive integration or plasticity disorders of the circuits (Lu et al., 2022). Therefore, the combined intervention strategy proposed in this review can be precisely implemented at the molecular, cellular, and systemic levels.

Future research should focus on the following conceptual breakthroughs: dynamically analyzing the interaction network between neurons and various glial cell subtypes in physiological and pathological states and elucidating the cell state space and its transition rules. The dynamic real-time cross-system dialogue (gut–brain and bone–brain axes) establishes a causal chain from peripheral signal changes to central coding rule changes. At the translational medicine level, gene therapy tools and closed-loop neuromodulation technology should be used to develop precision delivery systems that cross the BBB. The clinical paradigm transition from symptomatic treatment to systemic repair can only be realized through effective integration of basic mechanism analysis and engineering technology innovation.
